# A Novel Multi-Objective Trajectory Planning Method for Robots Based on the Multi-Objective Particle Swarm Optimization Algorithm

**DOI:** 10.3390/s24237663

**Published:** 2024-11-29

**Authors:** Jiahui Wang, Yongbo Zhang, Shihao Zhu, Junling Wang

**Affiliations:** 1 School of Aeronautic Science and Engineering, Beihang University, Beijing 100191, China; 2 Aircraft and Propulsion Laboratory, Ningbo Institute of Technology, Beihang University, Ningbo 315100, China; 3 School of Reliability and Systems Engineering, Beihang University, Beijing 100191, China

**Keywords:** Puma560 robot, multi-objective trajectory planning, MOPSO, B-spline

## Abstract

The three performance indexes of the space robot, travel time, energy consumption, and smoothness, are the key to its important role in space exploration. Therefore, this paper proposes a multi-objective trajectory planning method for robots. Firstly, the kinematics and dynamics of the Puma560 robot are analyzed to lay the foundation for trajectory planning. Secondly, the joint space trajectory of the robot is constructed with fifth-order B-spline functions, realizing the continuous position, velocity, acceleration, and jerk of each joint. Then, the improved multi-objective particle swarm optimization (MOPSO) algorithm is used to optimize the trajectory, and the distribution uniformity, convergence, and diversity of the obtained Pareto front are good. The improved MOPSO algorithm can realize the optimization between multiple objectives and obtain the trajectory that meets the actual engineering requirements. Finally, this paper implements the visualization of the robot’s joints moving according to the optimal trajectory.

## 1. Introduction

With the rapid development of science and technology in today’s world, humankind’s space exploration has shown unprecedented momentum, and space technology has gradually risen to the focus of public attention. Among them, space robots have become indispensable to space activities due to their powerful functions and high adaptability to the space environment. A series of challenging tasks, such as assembling satellite parts, capturing space targets, and monitoring alien spacecraft, cannot be realized without the support of space robots. These tasks and harsh working environments also set higher requirements for space robots’ travel time, energy consumption, and smoothness.

The primary method to improve each performance index is to design and plan the robot’s trajectory rationally. Trajectory planning can be performed both in Cartesian space and in joint space. The latter plans the trajectory of each joint of the robot, which has a small amount of calculation and enables the real-time control of the robot. Trajectory planning can usually be divided into two steps. The first step is to interpolate between given path points using interpolation algorithms to obtain a trajectory-time sequence. The second step is to optimize the trajectory in terms of single or multiple performance indexes within the constraints of the kinematics and dynamics of the robot [1].

The mainstream interpolation algorithms in joint space are polynomial interpolation and spline curve interpolation, and the former is mainly used in early research [2,3,4,5]. To obtain the robot’s trajectory, Ref. [6] used a cubic polynomial to connect the path points. This method is simple to calculate, but the acceleration curve obtained is not continuous, the smoothness could be better, and it tends to cause rigid impacts. Ref. [7] used quintic polynomial interpolation to ensure the continuity of acceleration, but it increased the amount of calculation, and there was still a problem of easy distortion. Compared to the fifth-degree polynomial, the seventh-degree polynomial adds constraints to the jerk at the start and termination points, realizing the continuity of the jerk. However, the eight boundary conditions increase the difficulty of the solution, and the high-order polynomial interpolation may cause the Runge phenomenon [8]. With the deepening of research, some scholars have applied spline curves to the trajectory planning of robots [9,10,11,12,13,14]. Ref. [15] used the fifth-order B-spline curves to interpolate the joint space trajectory, which realized the continuity of the jerk and set the velocity and acceleration at the start and stop time to be 0. When interpolating with seventh-order B-spline curves, it is possible to specify the acceleration at the start and stop time, but the calculation process is complicated [1].

Trajectory optimization mostly takes a single performance index as the optimization objective. Robot efficiency, the shortest time required by the robot to perform a task, was the earliest goal of trajectory planning [16,17,18,19]. Ref. [20] constructed the trajectory with a quintic polynomial and reduced the robot’s travel time by 74.35% through the improved MOPSO algorithm under the constraints of each joint’s angles, velocities, and accelerations. The time-optimal trajectory often leads to a large impact on the robot, affecting its motion accuracy and shortening the service life of the robot structure. Many scholars have solved this problem by optimizing the jerk. Ref. [7] effectively increased the smoothness of the robot by optimizing the maximum value of joint jerks. Ref. [21] combined the PSO algorithm with K-means clustering to achieve a fast solution for joint trajectories with minimal shocks. Energy consumption optimization is also an important issue for robots working in unique environments such as oceans, deserts, and space [22,23,24,25]. Ref. [26] obtained a parameterized dynamic robot model through identification experiments and used a sequential quadratic programming solver to minimize a mechanical energy-based cost function under consideration of physical constraints. These three performance indexes all play an important role in the motion of the space robot, and the single optimization objective ignores the intricate balance between them. Therefore, there are studies on the comprehensive optimization of multiple objectives [27,28,29]. Ref. [30] realized the comprehensive optimization of time, energy, and smoothness by a differential evolution algorithm. Ref. [31] used the NSGA-II algorithm to optimize the same three objectives and obtained Pareto optimal solution sets, thus obtaining the high-order continuous optimal trajectories.

There are few studies on multi-objective optimization problems, so this paper proposes a trajectory planning method that can make the robot’s travel time, energy consumption, and smoothness achieve the integrated optimal state when performing the task. In this paper, continuous and smooth joint space trajectories are constructed using fifth-order B-spline functions, which also realize the specification of the velocity and acceleration of the robot at the start/stop moment. The trajectories are then optimized using the improved MOPSO algorithm to obtain the Pareto optimal solution sets, from which suitable solutions are selected according to practical needs.

The rest of the paper is organized as follows. Section 2 analyzes the kinematics and dynamics of the Puma 560 robot manufactured by Unimation, USA. Section 3 uses fifth-order B-spline curves to construct the joint space trajectories of the robot, builds a mathematical model for the multi-objective optimization problem based on Section 2, and solves the model with the improved MOPSO algorithm. Section 4 performs simulation experiments on multi-objective trajectory planning. Section 5 summarizes this article.

## 2. Kinematics and Dynamics Analysis

### 2.1. Kinematics Analysis

**Figure 1:**
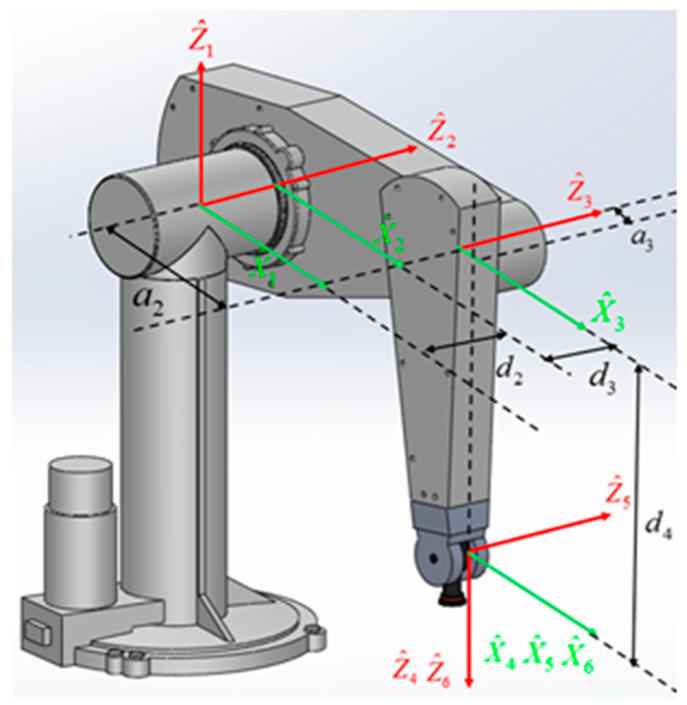
The MDH coordinate system of the Puma560 robot.

This section gives the kinematics model of the Puma560 robot and analyzes its forward and inverse kinematics. The MDH (Modified Denavit–Hartenberg) coordinate system [32] shown in Figure 1 is established on the Puma560 robot with six rotary joints. The link parameters from the MATLAB R2020b Robot Toolbox are shown in Table 1.

**Figure 2:**
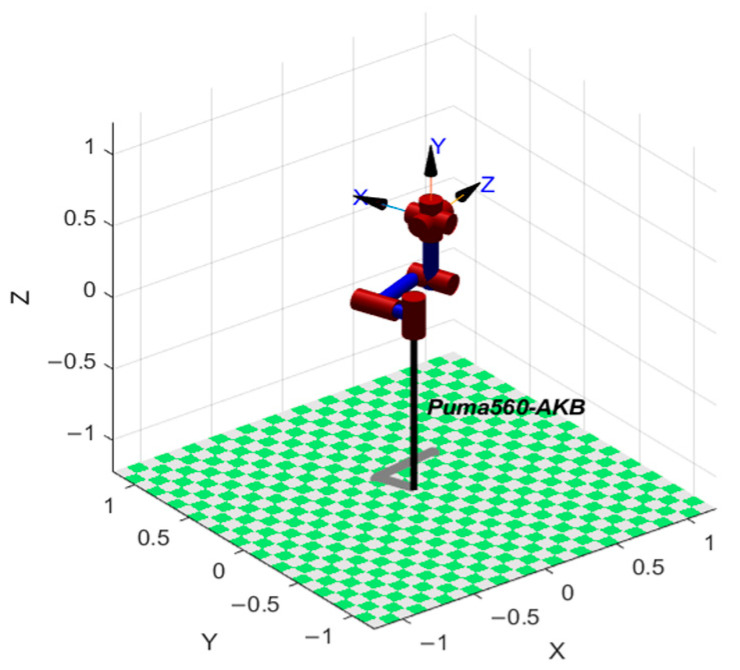
Robot model in MATLAB.

The simulation model of the Puma560 robot is established by using the Robot Toolbox in MATLAB, as shown in Figure 2.

#### 2.1.1. Forward Kinematics Analysis

Forward kinematics analysis refers to obtaining the end-effector pose relative to the base according to the angle of each robot joint. The transformation matrix between neighboring links, that is, the coordinate system {*i*} relative to the coordinate system {*i* − 1}, can be represented by

(1) Tii-1=cosθisinθicosαi−1sinθisinαi−10−sinθicosθicosαi−1cosθisinαi−100−sinαi−1cosαi−10ai−1−sinαi−1dicosαi−1di1

Substituting the parameters in Table 1 into (1), the six homogeneous transformation matrixes of the Puma560 robot can be obtained by

(2) T10=cosθ1−sinθ100sinθ1cosθ10000100001   T21=cosθ2−sinθ200001d2−sinθ2−cosθ2000001   T32=cosθ3−sinθ30a2sinθ3cosθ300001d30001T43=cosθ4−sinθ40a300−1−d4sinθ4cosθ4000001   T54=cosθ5−sinθ5000010−sinθ5−cosθ5000001   T65=cosθ6−sinθ60000−10sinθ6cosθ6000001.

The transformation matrixes in (2) can be multiplied together to find the transformation matrix of the end-effector coordinate system {6} relative to the base coordinate system {0}

(3) T60=T10T21T32T43T54T65=r11r21r310r12r22r320r13r23r330pxpypz1

where $p_{x},\;p_{y},\;p_{z}$ represent the position of the end-effector, and $r_{11},\;r_{12},\;r_{13},\;r_{21},\;r_{22},\;r_{23},\;r_{31},\;r_{32},\;r_{33}$ represent the orientation of the end-effector. These 12 elements are calculated by the joint angles.

#### 2.1.2. Inverse Kinematics Analysis

Inverse kinematics analysis refers to the inverse solution of the angle of each joint by using the end-effector pose relative to the base. Analyzing the structural characteristics of the Puma560 robot, it is easy to know that its last three axes intersect at one point, and the six joints of the robot are all rotary joints. So, the Pieper method [33] can solve the inverse kinematics of the Puma560 robot. When $\sin\left(\theta_{5}\right)\neq 0$, the joint angles can be obtained by

(4) $$\theta_{1}=A\tan2\left(\frac{g_{1}y-g_{2}x}{g_{1}^{2}+g_{2}^{2}},\frac{g_{1}x+g_{2}y}{g_{1}^{2}+g_{2}^{2}}\right)$$

(5) $$\theta_{2}=A\tan2\left(\frac{-z}{\rho_{2}},\pm \sqrt{1-\frac{z^{2}}{\rho_{2}^{2}}}\right)-A\tan2\left(f_{2},f_{1}\right)$$

(6) $$\theta_{3}=A\tan2\left(\frac{C-r}{\rho_{3}},\pm \sqrt{1-\frac{{\left(C-r\right)}^{2}}{\rho_{3}^{2}}}\right)-A\tan2\left(a_{3},d_{4}\right)$$

(7) $$\theta_{4}=A\tan2\left(\frac{x_{33}}{\sin(\theta_{5})},\frac{x_{13}}{\sin(\theta_{5})}\right)$$

(8) $$\theta_{5}=A\tan2\left(\pm \sqrt{x_{21}^{2}+x_{22}^{2}},-x_{23}\right)$$

(9) $$\theta_{6}=A\tan2\left(\frac{-x_{22}}{\sin(\theta_{5})},\frac{x_{21}}{\sin(\theta_{5})}\right)$$

where Atan2 is predefined in many programming language libraries; its function is to judge the quadrant of the angle according to the positive and negative of *x* and *y* while calculating $\tan^{-1}\left(\frac{y}{x}\right)$. Unknown in (4)–(9), such as $g_{1},\;g_{2}$, are the intermediate quantities in the derivation process of the Pieper method, which are defined as

(10) $$f_{1}=a_{2}+a_{3}c_{3}+d_{4}s_{3},\;f_{2}=a_{3}s_{3}-d_{4}c_{3},\;f_{3}=d_{3}$$

(11) $$g_{1}=f_{1}c_{2}-f_{2}s_{2},\;g_{2}=f_{3}+d_{2},\;g_{3}=-f_{1}s_{2}-f_{2}c_{2}$$

(12) $$r=f_{1}^{2}+f_{2}^{2}+f_{3}^{2}+d_{2}^{2}+2f_{3}d_{2},\;\;C=r-\left(a_{2}^{2}+a_{3}^{2}+d_{4}^{2}+d_{2}^{2}+d_{3}^{2}+2d_{2}d_{3}\right)$$

(13) $$x=\left(f_{1}c_{2}-f_{2}s_{2}\right)c_{1}-\left(f_{3}+d_{2}\right)s_{1},\;\;y=\left(f_{1}c_{2}-f_{2}s_{2}\right)s_{1}+\left(f_{3}+d_{2}\right)c_{1},\;\;z=-f_{1}s_{2}-f_{2}c_{2}$$

(14) $$\rho_{2}=\sqrt{f_{1}^{2}+f_{2}^{2}},\;\;\rho_{3}=2a_{2}\sqrt{a_{3}^{2}+d_{4}^{2}}$$

(15) $$\phi_{2}=A\tan2\left(f_{2},f_{1}\right),\;\;\phi_{3}=A\tan2\left(a_{3},d_{4}\right)$$

(16) $$x_{13}=r_{13}c_{23}c_{1}-r_{33}s_{23}+r_{23}c_{23}s_{1},\;\;x_{21}=-r_{31}c_{23}-r_{11}c_{1}s_{23}-r_{21}s_{1}s_{23}$$

(17) $$x_{22}=-r_{32}c_{23}-r_{12}c_{1}s_{23}-r_{22}s_{1}s_{23},\;\;x_{23}=-r_{33}c_{23}-r_{13}c_{1}s_{23}-r_{23}s_{1}s_{23},\;\;x_{33}=r_{23}c_{1}-r_{13}s_{1}$$

where $c_{i}=\cos\theta_{i},\;s_{i}=\sin\theta_{i},\;s_{ij}=\sin\left(\theta_{i}+\theta_{j}\right),\;c_{ij}=\cos\left(\theta_{i}+\theta_{j}\right),$ $i,j=\left[1,2,3,4,5,6\right]$, and i≠j.

If $\sin\left(\theta_{5}\right)\neq 0$ and $\theta_{5}=0$, then the solutions of $\theta_{1},\theta_{2},\theta_{3}$ do not change, and $\theta_{4},\theta_{5},\theta_{6}$ become

(18) $$\theta_{4}=0$$

(19) $$\theta_{5}=0$$

(20) $$\theta_{6}=A\tan2\left(-x_{12},x_{11}\right)$$

where $x_{11}=r_{11}c_{23}c_{1}-r_{31}s_{23}+r_{21}c_{23}s_{1},\;x_{12}=r_{12}c_{23}c_{1}-r_{32}s_{23}+r_{22}c_{23}s_{1}$.

If $\sin\left(\theta_{5}\right)\neq 0$ and $\theta_{5}=\pi$, then the solutions of $\theta_{1},\theta_{2},\theta_{3}$ do not change, and $\theta_{4},\theta_{5},\theta_{6}$ become

(21) $$\theta_{4}=0$$

(22) $$\theta_{5}=\pi$$

(23) $$\theta_{6}=A\tan2\left(x_{12},-x_{11}\right)$$

### 2.2. Dynamics Analysis

The iterative Newton–Euler dynamics algorithm [34] is computationally efficient, suitable for real-time control, and commonly used for modeling robot dynamics. The algorithm is composed of two parts. First, link velocities and accelerations are iteratively calculated from the base. Second, starting from the end-effector, the force and torque of each link are calculated in reverse. The specific iterative calculation process is as follows.

Outward iterations: $i:0\to n_{l}-1$

(24) wi+1i+1=Riii+1wi+θ˙i+1Z^i+1i+1

(25) w˙i+1i+1=Riii+1w˙i+Riii+1wi×θ˙i+1Z^i+1i+1+θ¨i+1Z^i+1i+1

(26) v˙i+1i+1=Rii+1w˙ii×Pi+1i+wii×wii×Pi+1i+v˙ii

(27) v˙Cii=w˙ii×PCii+wii×wii×PCii+v˙ii

(28) $$F_{i}=m{\dot{v}}_{C_{i}}$$

(29) Ni=ICiw˙i+wi×ICiwi

where $n_{l}$ is the number of links, Z^i+1i+1 is the unit vector of the coordinate system {*i* + 1} on the *Z* axis, $F_{i}$ and $N_{i}$ are, respectively, the inertia force and torque acting on the mass center of the link *i*.

Inward iterations: $i:n_{l}\to 1$

(30) fii=Ri+1i+1ifi+1+Fii

(31) nii=Nii+Ri+1i+1ini+1+PCii×Fii+Pi+1i×Ri+1i+1ifi+1

(32) τi=niTiZ^ii

where fii, nii are, respectively, the force and torque acting on the link *i*, and $\tau_{i}$ is the driving force of the joint motor.

## 3. Multi-Objective Trajectory Planning

### 3.1. Construction of Joint Space Trajectory

A *k*th-degree B-spline curve [35] is defined by

(33) $$p\left(u\right)=\sum_{i=0}^{n}d_{i}N_{i,k}\left(u\right)$$

where $d_{i}$ is the control point, *n*+1 is its number, $p\left(u\right)$ is the path point at node *u*, $N_{i,k}\left(u\right)$ is the *k*th-degree B-spline basis function, and its specific definition is

(34) $$\left\{\begin{array}{c} N_{i,0}\left(u\right)=\left\{\begin{array}{c} 1,\;\;u_{i}\leq u<u_{i+1}\\ 0,\;\;\mathrm{others} \end{array}\right.\\ N_{i,k}\left(u\right)=\frac{u-u_{i}}{u_{i+k}-u_{i}}N_{i,k-1}\left(u\right)+\frac{u_{i+k+1}-u}{u_{i+k+1}-u_{i+1}}N_{i+1,k-1}\left(u\right)\\ \frac{0}{0}=0. \end{array}\right.$$

The interval of $N_{i,k}\left(u\right)$, $u\in \left[u_{i},u_{i+k+1}\right]$, contains k+1 node intervals. It can be seen from (34) that for any node $u\in \left[u_{i},u_{i+k+1}\right]$ on the parameter axis, there are only up to k+1 nonzero basis functions $N_{r,k}\left(u\right)\left(r=i-k,i-k+1,\ldots ,i\right)$. This is the local support property of the B-spline curve. Therefore, the B-spline curve can also be expressed as

(35) $$p\left(u\right)=\sum_{r=i-k}^{i}d_{r}N_{r,k}\left(u\right)$$

In this paper, the joint space trajectory of the robot is obtained by fifth-order B-spline curve interpolation, so k=5. Assuming that the position-time series of a certain joint is $P=\left(p_{j},t_{j}\right),j=0,1,\ldots ,m$, then the node vector is $U=\left[u_{0},u_{1},\ldots ,u_{m+2k}\right]$, and n=m+k−1.

In order to make the B-spline curve pass through the first and end position points of the joint, the node repetition degree of these two positions needs to be defined as

(36) $$u_{0}=u_{1}=\ldots =u_{5}=0$$

(37) $$u_{m+5}=u_{n+6}=\ldots =u_{m+10}=1$$

Moreover, the accumulative chord length parameterization method normalizes the remaining m−1 inner nodes

(38) $$u_{i}=u_{i-1}+\frac{\left|\Delta t_{i-6}\right|}{\sum_{r=0}^{m-1}\left|\Delta t_{r}\right|},i=6,7,\ldots ,m+4$$

The n+1 equations are needed to solve n+1 control points, where m+1 equations can be given by

(39) $$p\left(u_{i+5}\right)=\sum_{r=i}^{i+5}d_{r}N_{r,5}\left(u_{i+5}\right)=p_{i},\;\;u_{i+5}\in \left[u_{5},u_{m+5}\right],i=0,1,\ldots ,m$$

Additional conditions determine the other k−1 equations. For the fifth-order B-spline curve, specifying the velocity and acceleration of the joint at the start and end points can add four additional equations

(40) $$\left\{\begin{array}{l} p^{\prime }\left(u\right){|}_{u=u_{5}}=v_{s},\;\;p^{\prime }\left(u\right){|}_{u=u_{m+5}}=v_{e}\\ p^{\prime \prime }\left(u\right){|}_{u=u_{5}}=a_{s},\;\;p^{\prime \prime }\left(u\right){|}_{u=u_{m+5}}=a_{e} \end{array}\right.$$

where $p^{\prime }\left(u\right),\;p^{\prime \prime }\left(u\right)$ are, respectively, the first and second derivatives of the B-spline curve, representing the velocity and acceleration of the joint. The deBoor–Cox recurrence formula can calculate the *l*th derivative of the B-spline curve

(41) $$\left\{\begin{array}{c} p^{l}\left(u\right)=\sum_{r=i-k+l}^{i}d_{r}^{l}N_{r,k-l}\left(u\right),\;\;u_{i}\leq u<u_{i+1}\\ d_{r}^{l}=\left\{\begin{array}{c} d_{j},\;\;l=0\\ \left(k+1-l\right)\left(d_{r}^{l-1}-d_{r-1}^{l-1}\right)/\left(u_{r+k+1-l}-u_{r}\right),\;\;l=1,2,\ldots ,r. \end{array}\right. \end{array}\right.$$

Expression (39) is combined with (40) to obtain

(42) $$A_{n}d=p$$

where $d={\left[d_{0},d_{1},\ldots ,d_{n-1},d_{n}\right]}^{T},\;p={\left[p_{0},p_{1},\ldots ,p_{m},v_{s},v_{e},a_{s},a_{e}\right]}^{T}$, and the coefficient matrix is

$$A_{n}=\left[\begin{array}{ccccccccc} 1 & & & & & & & & \\ & N_{1,5}(u_{6}) & N_{2,5}(u_{6}) & \cdots & N_{5,5}(u_{6}) & & & & \\ & & N_{2,5}(u_{7}) & N_{3,5}(u_{7}) & \cdots & N_{6,5}(u_{7}) & & & \\ & & \ddots & & & \ddots & & & \\ & & & N_{m-2,5}(u_{m+3}) & N_{m-1,5}(u_{m+3}) & \cdots & N_{m+2,5}(u_{m+3}) & & \\ & & & & N_{m-1,5}(u_{m+4}) & N_{m,5}(u_{m+4}) & \cdots & N_{m+3,5}(u_{m+4}) & \\ & & & & & & & & 1\\ c_{s1} & c_{s2} & & & & & & & \\ & & & & & & & c_{e1} & c_{e2}\\ a_{s1} & a_{s2} & & & & & & & \\ & & & & & & a_{e1} & a_{e2} & a_{e3} \end{array}\right]$$

Some parameters in the coefficient matrix are defined by

(43) $$\begin{array}{c} c_{s1}=-5/\left(u_{6}-u_{1}\right)\\ c_{s2}=5/\left(u_{6}-u_{1}\right)\\ c_{e1}=-5/\left(u_{m+9}-u_{m+4}\right)\\ c_{e2}=5/\left(u_{m+9}-u_{m+4}\right)\\ a_{s1}=20/\left[\left(u_{6}-u_{2}\right)\left(u_{6}-u_{1}\right)\right]\\ a_{s2}=-20\left\{1/\left[\left(u_{6}-u_{2}\right)\left(u_{6}-u_{1}\right)\right]+1/\left[\left(u_{6}-u_{2}\right)\left(u_{7}-u_{2}\right)\right]\right\}\\ a_{s3}=20/\left[\left(u_{6}-u_{2}\right)\left(u_{7}-u_{2}\right)\right]\\ a_{e1}=20/\left[\left(u_{m+8}-u_{m+4}\right)\left(u_{m+8}-u_{m+3}\right)\right]\\ a_{e2}=-20\left\{1/\left[\left(u_{m+8}-u_{m+4}\right)\left(u_{m+8}-u_{m+3}\right)\right]+1/\left[\left(u_{m+8}-u_{m+4}\right)\left(u_{m+9}-u_{m+4}\right)\right]\right\}\\ a_{e3}=20/\left[\left(u_{m+8}-u_{m+4}\right)\left(u_{m+9}-u_{m+4}\right)\right]. \end{array}$$

From (42), all the control points can be obtained by

(44) $$d=A_{n}^{-1}p$$

Bringing the control points back to (35), the angular displacement curve of each robot joint can be obtained. Then, the angular velocity, angular acceleration, and angular jerk curves of each joint are obtained by (41).

### 3.2. Establishing the Multi-Objective Optimization Model

This model consists of objective functions and constraint conditions. Firstly, the specific expressions of travel time, energy consumption, and smoothness are shown in (45)–(47). It is assumed that the trajectory of each joint of the Puma560 is divided into *m* segments, which means that each joint is assigned *m* + 1 angle values in turn.

The total travel time of the robot is the sum of the travel time of each trajectory, so its expression is

(45) $$S_{1}=T=\sum_{j=1}^{m}\Delta t_{j}=\sum_{j=1}^{m}\left(t_{j}-t_{j-1}\right)$$

where $t_{j-1},\;t_{j},\;\Delta t_{j}$ are, respectively, the starting time, ending time, and travel time of the *j*th trajectory, and T is the total travel time.

The average accelerations of joints represent the energy consumption

(46) $$S_{2}=\sum_{i=1}^{6}\sqrt{\frac{1}{T}\int_{0}^{T}{\ddot{\theta }}_{i}^{2}dt}$$

where ${\ddot{\theta }}_{i}$ is the acceleration of the *i*th joint.

The average jerks of joints measure the smoothness

(47) $$S_{3}=\sum_{i=1}^{6}\sqrt{\frac{1}{T}\int_{0}^{T}{\dddot{\theta }}_{i}^{2}dt}$$

where ${\dddot{\theta }}_{i}$ is the jerk of the *i*th joint.

Constraints of kinematics and dynamics of the robot need to be considered when the robot performs tasks. Kinematic constraints mainly include the limitation of joint angle, velocity, and acceleration. Dynamic constraints mostly refer to the restriction of the joint torque. Therefore, the multi-objective optimization model can be established as

(48) $$\begin{array}{l} \mathrm{Minimize}\\ \;\;\;S={\left[S_{1},S_{2},S_{3}\right]}^{T}\\ \mathrm{Subject}\;\mathrm{to}\;\\ g_{i,1}=\theta_{i}^{\min}-\min\left(\theta_{i}\left(t\right)\right)\leq 0\\ g_{i,2}=\max\left(\theta_{i}\left(t\right)\right)-\theta_{i}^{\max}\leq 0\\ g_{i,3}={\dot{\theta }}_{i}^{\min}-\min\left({\dot{\theta }}_{i}\left(t\right)\right)\leq 0\\ g_{i,4}=\max\left({\dot{\theta }}_{i}\left(t\right)\right)-{\dot{\theta }}_{i}^{\max}\leq 0\\ g_{i,5}={\ddot{\theta }}_{i}^{\min}-\min\left({\ddot{\theta }}_{i}\left(t\right)\right)\leq 0\\ g_{i,6}=\max\left({\ddot{\theta }}_{i}\left(t\right)\right)-{\ddot{\theta }}_{i}^{\max}\leq 0\\ g_{i,7}=\tau_{i}^{\min}-\min\left(\tau_{i}\left(t\right)\right)\leq 0\\ g_{i,8}=\max\left(\tau_{i}\left(t\right)\right)-\tau_{i}^{\max}\leq 0. \end{array}$$

where $\theta_{i}\left(t\right),\;{\dot{\theta }}_{i}\left(t\right),\;{\ddot{\theta }}_{i}\left(t\right),\;\tau_{i}\left(t\right)$ are, respectively, the angle, velocity, acceleration, and driving torque of the *i*th joint at *t* time, and $\theta_{i}^{\max},\;\theta_{i}^{\min},\;{\dot{\theta }}_{i}^{\max},\;{\dot{\theta }}_{i}^{\min},\;{\ddot{\theta }}_{i}^{\max},\;{\ddot{\theta }}_{i}^{\min},\;\tau_{i}^{\max},\;\tau_{i}^{\min}$ are, respectively, the upper and lower limits of the angle, velocity, acceleration, and driving torque of the *i*th joint.

### 3.3. Solving the Multi-Objective Optimization Model

The three optimization objectives conflict with each other, and there is a complex balance between them, so they cannot achieve the best solution simultaneously. When the improved MOPSO algorithm is used to solve the previous model, the result is no longer a single optimal solution, but an optimal solution set called the Pareto optimal solution set [36]. There is no good or bad solution in the Pareto optimal solution set, and the appropriate solution can be selected according to the actual engineering needs.

The MOPSO algorithm [37] originated from the study of bird foraging behavior and has the advantages of fast convergence speed, strong global search capability, and a wide range of application. In this algorithm, each particle’s position represents a solution to the problem. Under the influence of the individual optimal position and group optimal position, particles update their velocity and position

(49) $$v_{id}^{k+1}=wv_{id}^{k}+c_{1}r_{1}\left(p_{id,pbest}^{k}-x_{id}^{k}\right)+c_{2}r_{2}\left(p_{d,gbest}^{k}-x_{id}^{k}\right)$$

(50) $$x_{id}^{k+1}=x_{id}^{k}+v_{id}^{k+1}$$

where w is the inertia weight, $c_{1}$ and $c_{2}$ are the individual and group learning factors, $r_{1}$ and $r_{2}$ are random numbers in the range of [0,1], k is the current iteration number, d is the vector’s dimension number, $x_{id}^{k}$ and $v_{id}^{k}$ are the position and velocity of particle *i*, and $p_{id,pbest}^{k}$ and $p_{d,gbest}^{k}$ are the optimal position of individual and group.

Furthermore, the algorithm determines the dominance relationship between particles by comparing the objective function values of particles. To better manage and preserve the non-dominated solution, it is saved to the external archive.

The traditional MOPSO algorithm determines the global leader and deletes the redundant particles in the external archive by random selection. The convergence, distribution uniformity, and accuracy of the Pareto front are not good. When facing complex problems, the algorithm easily falls into the local optimum.

**Figure 3:**
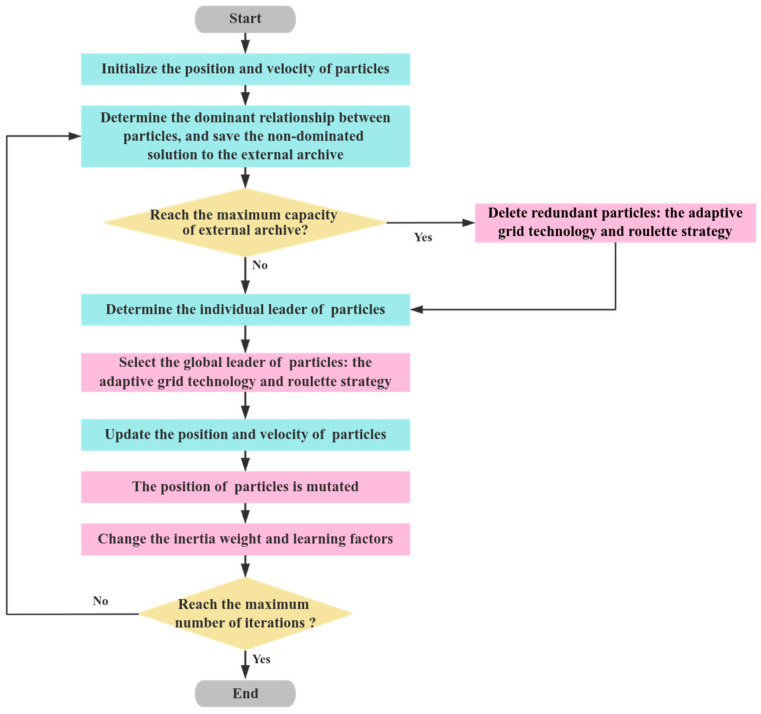
The improved MOPSO algorithm.

In order to solve these problems, this paper uses the adaptive grid technology and roulette strategy to change the selection of the global leader and redundant particles in the external archive, applies the adaptive mutation technique to the position of particles, and makes the inertia weight, individual, and group learning factors change nonlinearly with the iterations number. The workflow flow chart of the improved MOPSO algorithm is show in Figure 3.

The combination of the adaptive grid technology and roulette strategy enables the algorithm to select particles in a suitable sub-grid. For each iteration, the maximum value $f_{i\max}$ and minimum value $f_{i\min}$ of the *i*th objective function on all particles are found as the upper and lower bounds of the initial grid. In order to cover the boundary particles, the range of the grid is expanded according to

(51) $$LB_{i}=f_{i\min}-\alpha \left(f_{i\max}-f_{i\min}\right),UB_{i}=f_{i\max}+\alpha \left(f_{i\max}-f_{i\min}\right)$$

where $LB_{i},UB_{i}$ are the new upper and lower bounds of the grid, and α is the expansion ratio.

**Figure 4:**
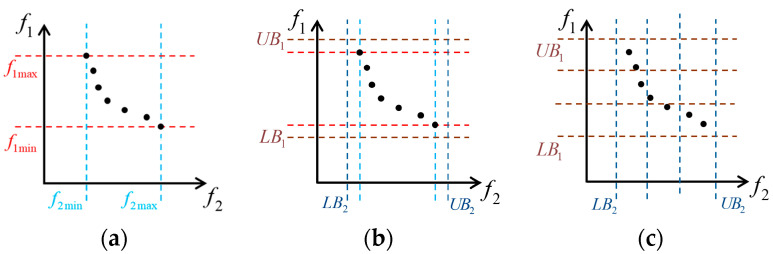
The adaptive grid technology in two-dimensional case. (**a**) The minimum and maximum values of each objective function; (**b**) the enlarged grid range; (**c**) the uniform distribution of grids when $n_{d}=3$.

The number of grids for each dimension is set to $n_{d}$, and the grid is equally divided into $n_{d}^{e}$ sub-grids, where e is the number of objective function. Figure 4 shows the general process of adaptive grid technology.

Suppose that there are $n_{s}$ sub-grids containing particles, and the number of particles in the *i*th sub-grid is $N_{i}$. When selecting the global leader of particles, the probability that the *i*th sub-grid is selected is

(52) $$P_{i,1}=\frac{e^{-\beta N_{i}}}{e^{-\beta N_{1}}+e^{-\beta N_{2}}+\cdots +e^{-\beta N_{b}}}$$

where $i=1,2,\ldots ,n_{s}$, and β is a non-negative leader selection pressure parameter. It can be seen from (52) that the global leader of particles is more likely to come from sub-grids with fewer particles, which encourages the algorithm to explore areas that have been searched less before and increase the diversity of solutions.

When the number of particles in the external archive is greater than the set number, it is necessary to delete the redundant particles. At this time, the probability that the *i*th sub-grid is selected is

(53) $$P_{i,2}=\frac{e^{\sigma N_{i}}}{e^{\sigma N_{1}}+e^{\sigma N_{2}}+\cdots +e^{\sigma N_{b}}}$$

where σ is a non-negative delete selection pressure parameter. It can be seen from (63) that the deleted redundant particles are more likely to come from the sub-grids with more particles, promoting the uniform distribution of the solutions.

Expressions (52)–(53) are the individual selection probabilities in the roulette strategy, and the cumulative probabilities of each grid are defined as

(54) $$Q_{i,1}=\sum_{j=1}^{i}P_{j,1},\;Q_{i,2}=\sum_{j=1}^{i}P_{j,2}.$$

The individual selection strategy is to generate a random number $r\in \left[0,1\right]$, compare it with $Q_{i,1}$ or $Q_{i,2}$, find the first cumulative probability exceeding r, and select its sub-grid.

When the particle’s velocity and position are updated, the particle’s position is adaptively mutated. The mutation rate in the *k*th iteration is

(55) $$pm_{k}={\left(1-\frac{k-1}{M-1}\right)}^{\frac{1}{h}}$$

where k is the current iteration number, M is the maximum iteration number, and *h* is a given constant.

The mutation step of the particle *i* is defined as

(56) $$\Delta x_{i}^{k}=pm_{k}\cdot \left(x_{\max}-x_{\min}\right)$$

where $x_{\max},x_{\min}$ are the limit values of particle position. When $pm_{k}$ decreases nonlinearly with the increase in the iterations number, $\Delta x_{i}^{k}$ also decreases.

Now, it is randomly specified that the *d*th dimensional component $x_{id}^{k}$ of the position vector is mutated. The upper and lower bounds of the range of mutation are

(57) $$lb=x_{id}^{k}-\Delta x_{i}^{k},ub=x_{id}^{k}+\Delta x_{i}^{k}$$

A random number is generated in the continuous distribution of lb and ub as the value of the position vector after mutation in this dimension. This makes the algorithm jump out of the local optimum in the early stage and converge to the global optimal solution better in the later stage.

The relationship between the inertia weight and the number of iterations is

(58) $$w=w_{\max}-\left(w_{\max}-w_{\min}\right){\left(\frac{k}{M}\right)}^{2}$$

where $w_{\max},w_{\min}$ are the upper and lower limits of w. As the number of iterations k increases, the inertia weight decreases nonlinearly.

The individual and group learning factors are defined as

(59) $$c_{1}=c_{1s}+\left(c_{1e}-c_{1s}\right)\sin\left(\frac{\pi k}{2M}\right),c_{2}=c_{2s}+\left(c_{2e}-c_{2s}\right)\sin\left(\frac{\pi k}{2M}\right)$$

where $c_{1s}$ and $c_{2s}$ are, respectively, the initial values of $c_{1}$ and $c_{2}$, and $c_{1e}$ and $c_{2e}$ are, respectively, the final values of $c_{1}$ and $c_{2}$. As the number of iterations increases, $c_{1}$ decreases from large to small, and $c_{2}$ is the opposite.

These give the particle a strong global search capability at the beginning of the algorithm iterations to avoid falling into local optimum and a strong local search capability at the later stages to improve convergence accuracy.

## 4. Simulation

In this section, the multi-objective trajectory planning simulation of the Puma560 robot is carried out in MATLAB. The constraints of each joint are shown in Table 2.

The target captured by the robot is a small ball moving at a constant speed of 0.5 m/s along the *Z*-axis, and its trajectory is known. Six key positions of each joint in the process of catching the ball are given, as shown in Table 3.

The MOPSO algorithm takes the time interval of each trajectory $\Delta t_{j}$ as the decision variable, which is in the range of [0.75, 7]. The population size and the maximum iteration number of the traditional and improved MOPSO algorithms are both 200, and the size of the Pareto optimal solution set is 100. In addition, this paper sets $n_{d}$ in the improved MOPSO algorithm to 5, β to 2, and σ to 2.

**Figure 5:**
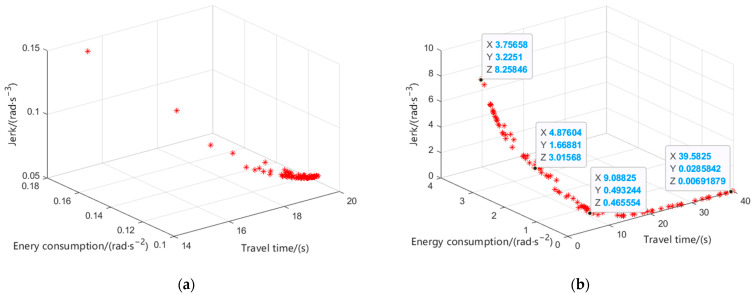
The Pareto front. (**a**) The traditional MOPSO algorithm and (**b**) the improved MOPSO algorithm.

The Pareto front obtained by the traditional MOPSO algorithm is shown in Figure 5a. It falls into the local optimum, and the distribution and convergence of the Pareto front are also poor. The Pareto front obtained by the improved MOPSO algorithm is shown in Figure 5b. It jumps out of the local optimum, and the convergence and distribution are significantly improved, which proves the effectiveness of the improved MOPSO algorithm.

Four points are taken on the Pareto front, which, from top to bottom, are A, B, C, and D. The closer to A, the shorter the travel time, the more the energy consumption, and the greater the jerk; the closer to D, the less the energy consumption, the smaller the jerk, and the longer the travel time. It follows that smoothness is positively correlated with energy consumption, while they are negatively correlated with travel time. The values of the three objective functions for A, B, C, and D are shown in Table 4.

Taking B and C points as examples, the travel time of point B is 4.8760 s, 46.35% less than that of point C. The energy consumption of point C is 0.4932 $\mathrm{rad}\cdot s^{-2}$, 70.45% lower than that of point B. The jerk of point C is 0.4656 $\mathrm{rad}\cdot s^{-3}$, 84.56% lower than that of point B.

**Figure 6:**
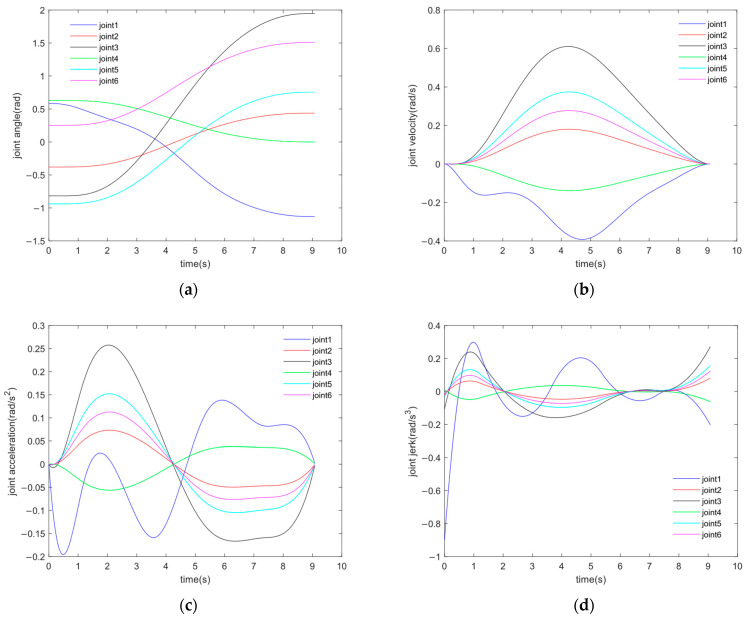
(**a**–**d**) are the angle, velocity, acceleration, and jerk curves of the joints under solution C.

C is selected as the actual solution of the project, and its time series is [0, 1.1990, 3.6445, 5.3612, 7.2749, 9.0883]. As shown in Figure 6, the curves of joint angles, velocities, accelerations, and jerks varying with time can be obtained by interpolating fifth-order B-spline curves.

A dominant solution E is randomly selected outside the Pareto optimal solution set as the time series [0, 1.3, 2.4, 5.3, 8.4, 10.4] before the trajectory optimization. Under this time series, the three performance indexes of the robot are shown in Table 5.

According to the data in Table 5, the travel time of point C is 12.61% less than that of E, the energy consumption is decreased by 56.95%, and the jerk is decreased by 75.15%. The three objective function values of point C are better than the results before optimization, improving the robot’s comprehensive performance.

**Figure 7:**
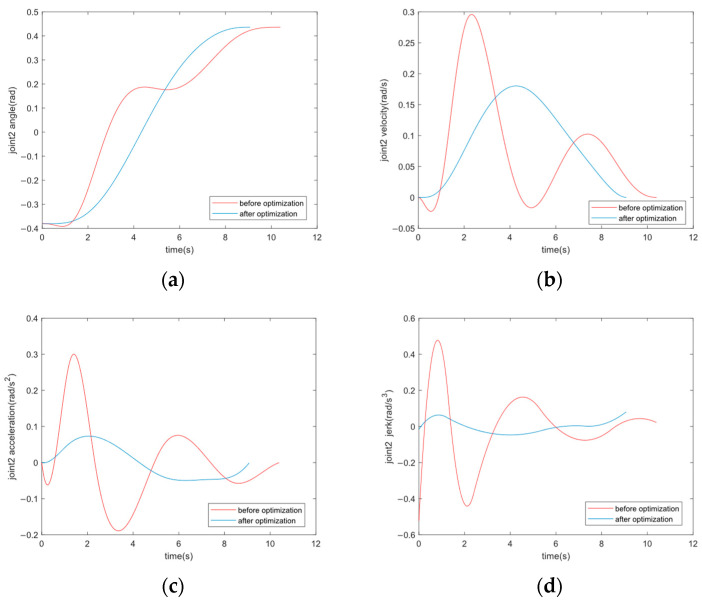
(**a**–**d**) show the curves of angle, velocity, acceleration, and jerk of robot joint 2 before and after optimization.

Taking robot joint 2 as an example, Figure 7 shows that the trajectories after optimization are smoother and more continuous than before optimization, especially the curves of joint velocity, acceleration, and jerk.

**Figure 8:**
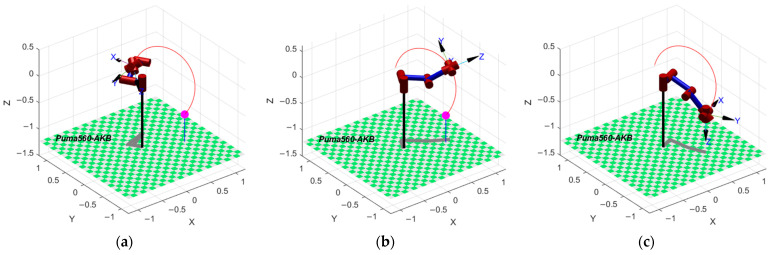
(**a**) The pose of the robot at the start moment; (**b**) the pose of the robot at the middle moment; and (**c**) the pose of the robot at the end moment.

An optimal solution is randomly selected in the Pareto optimal solution set, called D, and its time series is [0, 1.7255, 4.5821, 6.1810, 7.7798, 10.0000]. The motion of the Puma560 robot under this solution can be visualized by the Robot Toolbox of MATLAB, as shown in Figure 8.

## 5. Conclusions

This paper investigates a trajectory planning method for a robot which enables it to reach a comprehensive optimal state of travel time, energy consumption, and smoothness when executing a task. In order to fully understand the kinematics and dynamics characteristics of the robot and lay a solid theoretical foundation for follow-up research, this paper first deduces the position and orientation of the end-effector relative to the base and uses the Pieper method to calculate the closed solutions of the inverse kinematics. Finally, the dynamic model of the robot is established by the iterative Newton–Euler dynamics algorithm.

The joint space trajectory of the Puma560 robot is constructed using fifth-order B-spline curves, which has the advantages of continuous jerk and zero velocity and acceleration at the start/stop time. Then, the improved MOPSO algorithm is used to optimize the trajectory of the robot with the time interval between the path points as the decision variable. The convergence and distribution of the Pareto front are good, and the different solutions in the Pareto optimal solution set correspond to different engineering needs. In addition, by comparing the robot’s travel time, energy consumption, and smoothness before and after optimization, it can be seen that its three performances have improved. This paper also visualizes the robot movement according to the planned trajectory in the Robot Toolbox of MATLAB.

## Figures and Tables

**Table 1:** Link parameters of the Puma560 robot.

Link *i*	$\alpha_{i-1}$ (rad)	$a_{i-1}$ (m)	$d_{i}$ (m)	$\theta_{i}$ (rad)
1	0	0	0	$\theta_{1}$
2	−1.5708	0	0.2435	$\theta_{2}$
3	0	0.4318	−0.0934	$\theta_{3}$
4	1.5708	−0.0203	0.4331	$\theta_{4}$
5	−1.5708	0	0	$\theta_{5}$
6	1.5708	0	0	$\theta_{6}$

**Table 2:** Kinematic and dynamic constraints.

Constraints	Joint 1	Joint 2	Joint 3	Joint 4	Joint 5	Joint 6
Angle/(rad)	3.100	3.100	3.100	3.100	3.100	3.100
$\mathrm{Velocity}/(\mathrm{rad}\cdot s^{-1}$)	0.876	0.876	1.598	0.876	0.926	0.926
$\mathrm{Acceleration}/(rad\cdot s^{-2}$)	0.725	0.725	2.378	0.725	1.450	1.450
Torque/(N⋅m)	44.940	44.940	8.866	44.940	0.050	0.050

**Table 3:** Position sequence of each joint.

Node	Joint 1/(rad)	Joint 2/(rad)	Joint 3/(rad)	Joint 4/(rad)	Joint 5/(rad)	Joint 6/(rad)
1	0.5821	−0.3805	−0.8168	0.6283	−0.9390	0.2531
2	0.4829	−0.3735	−0.7981	0.6299	−0.9245	0.2621
3	0.0383	−0.1212	0.0608	0.4289	−0.4005	0.6502
4	−0.5872	0.1770	1.0702	0.1995	0.2189	1.1091
5	−1.0317	0.3890	1.7877	0.0364	0.6592	1.4352
6	−1.1310	0.4363	1.9478	0	0.7547	1.5080

**Table 4:** The partial optimum solution.

Solution	Travel Time/(s)	$\mathrm{Energy}\;\mathrm{Consummation}/(rad\cdot s^{-2}$)	$\mathrm{Jerk}/(rad\cdot s^{-3}$)
A	3.7566	3.2251	8.2585
B	4.8760	1.6688	3.0157
C	9.0883	0.4932	0.4656
D	39.5825	0.0286	0.0069

**Table 5:** Comparison before and after optimization.

Solution	Travel Time/(s)	$\mathrm{Energy}\;\mathrm{Consumption}/(rad\cdot s^{-2}$)	$\mathrm{Jerk}/(rad\cdot s^{-3})$
C	9.0883	0.4932	0.4656
E	10.4000	1.1457	1.8733

## Data Availability

Data are contained within the article.
